# The Cell Division Cycle of *Euglena gracilis* Indicates That the Level of Circadian Plasticity to the External Light Regime Changes in Prolonged-Stationary Cultures

**DOI:** 10.3390/plants10071475

**Published:** 2021-07-19

**Authors:** Shota Kato, Hong Gil Nam

**Affiliations:** 1 Center for Plant Aging Research, Institute for Basic Science, Daegu 42988, Korea; 2 Department of New Biology, DGIST, Daegu 42988, Korea

**Keywords:** aging, cell division, circadian plasticity, *Euglena gracilis*, microalgae, T-cycle

## Abstract

In unicellular photosynthetic organisms, circadian rhythm is tightly linked to gating of cell cycle progression, and is entrained by light signal. As several organisms obtain a fitness advantage when the external light/dark cycle matches their endogenous period, and aging alters circadian rhythms, senescence phenotypes of the microalga *Euglena gracilis* of different culture ages were characterized with respect to the cell division cycle. We report here the effects of prolonged-stationary-phase conditions on the cell division cycles of *E. gracilis* under non-24-h light/dark cycles (T-cycles). Under T-cycles, cells established from 1-month-old and 2-month-old cultures produced lower cell concentrations after cultivation in the fresh medium than cells from 1-week-old culture. This decrease was not due to higher concentrations of dead cells in the populations, suggesting that cells of different culture ages differ in their capacity for cell division. Cells from 1-week-old cultures had a shorter circadian period of their cell division cycle under shortened T-cycles than aged cells. When algae were transferred to free-running conditions after entrainment to shortened T-cycles, the young cells showed the peak growth rate at a time corresponding to the first subjective night, but the aged cells did not. This suggests that circadian rhythms are more plastic in younger *E. gracilis* cells.

## 1. Introduction

A wide variety of organisms manifest circadian biological rhythms in order to anticipate and prepare for environmental changes caused by Earth’s rotation. In land plants, endogenous biological clocks adjust the timing of developmental, physiological, metabolic, and reproductive processes—such as hypocotyl elongation, stomatal opening, photosynthesis, and flowering—to the proper time of day and season (for review, see [1,2]). Unicellular organisms have also developed circadian clocks [3]. In both prokaryotic cyanobacteria and eukaryotic microalgae—such as *Synechococcus elongatus*, *Chlamydomonas reinhardtii*, *Cyanidioschyzon merolae*, and *Euglena gracilis*—circadian rhythms are tightly linked to the gating of cell cycle progression, which allows cells to divide only at certain circadian timings [4,5,6,7].

The clock oscillation is sensitive and entrained to environmental cues, such as light and temperature. Recent findings revealed that the clocks in cells within an individual plant run at different phases, periods, amplitudes, and robustness of oscillations, depending on tissues and organs in response to their microenvironment [8,9,10,11,12]. In addition, circadian rhythms in plants alter with aging. The term aging can be defined as the addition of time to cells, tissues, organs, and organisms [13]. Aging is typically associated with cellular senescence—a hallmark of aged cells. Senescence is accompanied by the global deterioration of functions and structures of cells, such as the dismantling of chloroplasts, loss of photosynthetic performance, and hydrolysis of macromolecules [14,15]. Working with *Arabidopsis thaliana*, Kim et al. [16] revealed that, within an individual plant, early-emerging (older) leaves had shorter circadian periods of core clock oscillator genes than late-emerging (younger) leaves. Other studies showed that—in cyanobacteria, land plants, and insects—organisms gain a fitness advantage when the external light/dark cycle (T-cycle) matches the endogenous period—a phenomenon known as “circadian resonance” [17,18,19].

The Euglenida, a group of photosynthetic unicellular eukaryotes [20], are among the model organisms most commonly used for studies of biochemistry, cellular and molecular biology, tactic responses (including phototaxis and gravitaxis), and the cell division cycle [21,22]. Although circadian rhythms of the physiology and biochemistry of *E. gracilis* have been investigated in detail over several decades [5,23,24], it remains unknown whether they are altered by aging in this alga. We therefore characterized the senescent phenotypes of *E. gracilis*—a model unicellular photosynthetic eukaryote—with respect to the circadian rhythm of cell division. Previous studies of tissue-/organ-specific flexibility in clock oscillations and age-dependent alterations in circadian rhythms led us to hypothesize that the circadian rhythm of *E. gracilis* would differ depending on the age of the culture. To test this, we measured the cell division cycle period in *E. gracilis* populations of different culture ages in batch culture, as well as its responsiveness to non-24 h light/dark cycles (T-cycles). *E. gracilis* cells pre-cultured for 1 week (young cells) before transfer to 8 h/8 h light/dark cycles (T16 cycles) divided partly in response to the external light/dark cycle, whereas cells pre-cultured for 1 or 2 months (aged cells) divided with a period of 32 h, indicating frequency demultiplication. When the algae were transferred to free-running conditions (high-frequency light/dark cycles) after entrainment to T16 cycles, the timing of the peak growth rate of young cells corresponded with the first subjective night, but that of aged cells did not. These results suggest that the circadian rhythms of young cells of *E. gracilis* have a higher level of plasticity, and are more responsive to the external environment, enabling young cells to synchronize their endogenous period of cell division with the external light regime.

## 2. Results

### 2.1. Characteristics of E. gracilis Cells in Old Cultures

**Figure 1:**
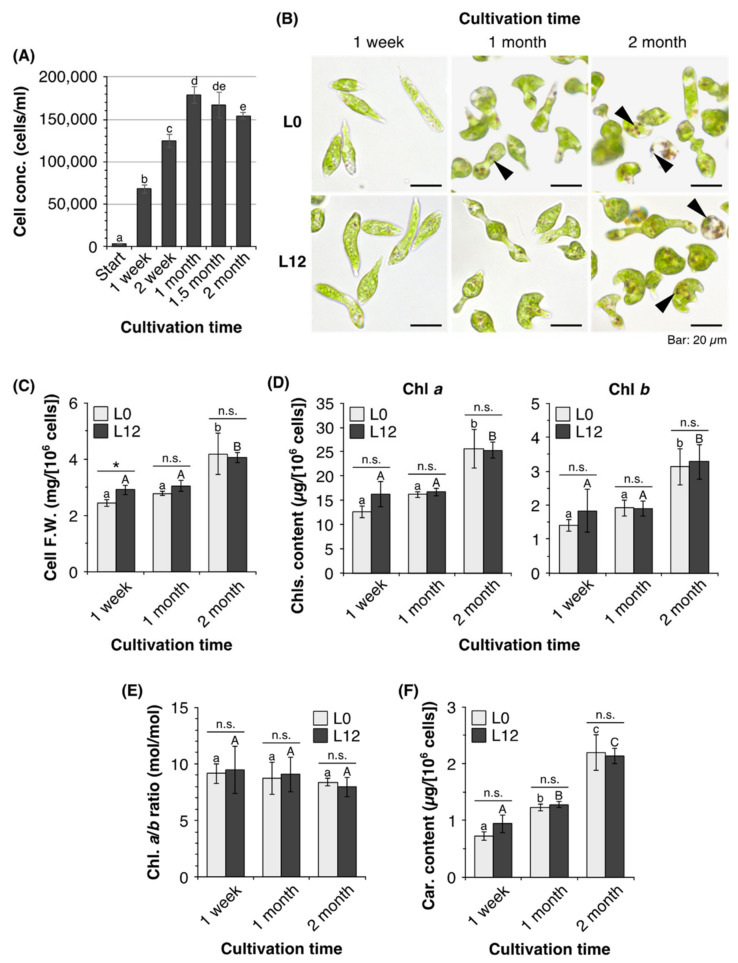
Characterization of *Euglena gracilis* cells in cultures of different ages. (**A**) Time course of cell concentrations of *E. gracilis* cells grown under 12 h/12 h light/dark cycles for 2 months. Data are means ± SD (*n* = 5). (**B**) Appearance of *E. gracilis* cells grown under 12 h/12 h light/dark cycles for 2 months. Algal cells were observed under a bright-field microscope at the beginning (L0) and the end (L12) of the light period after 1 week, 1 month, and 2 months in culture. Arrowheads indicate pigmented brown globules within cells. (**C**–**F**): (**C**) Cell fresh weight; (**D**) chlorophyll *a* and *b* content; (**E**) chlorophyll *a*:*b* ratio; and (**F**) total carotenoid content of cells from 1-week-old and 1- or 2-month-old cultures. Data are means ± SD (*n* = 3). Bars labeled with the same letter do not differ significantly from one another (Tukey’s multiple range test, *p* < 0.05). n.s.: not significant; *: *p* < 0.05 (*t*-test).

To characterize senescent phenotypes of *E. gracilis* in prolonged stationary phase, we cultured algal cells for 2 months without either replacing or supplementing the medium. When *E. gracilis* cells were grown under 12 h/12 h light/dark cycles at 25 °C, the cell concentration increased to 1.8 × 10^5^ cells mL^–1^ after 1 month (Figure 1A). Subsequently, the cell concentration gradually decreased as the time in culture increased until, after cultivation for 2 months, it had decreased by 14% compared with a 1-month-old culture. Bright-field microscopy showed that cells from 1- or 2-month-old cultures differed from those from 1-week-old cultures (Figure 1B). Algal cells from 1-week-old cultures had elongated cylindrical or spindle shapes, whereas most cells from 1- or 2-month-old cultures exhibited gourd or mushroom shapes. In addition, cells from 1- or 2-month-old cultures contained several brown globules. The cells from 2-month-old cultures appeared larger than those from 1-week-old cultures and, indeed, the fresh weights of cells from 2-month-old cultures were 1.7 and 1.4 times higher than those of cells from 1-week-old cultures at the beginning (L0) and end (L12) of the light period, respectively (Figure 1C).

We next measured the photosynthetic pigment (chlorophylls and carotenoids) content of *E. gracilis* cells after 1 week, 1 month, and 2 months in culture. The Chl *a* content of cells from 2-month-old cultures was 2-fold and 1.6-fold higher at L0 and L12, respectively, than that of cells from 1-week-old cultures (Figure 1D). Similarly, cells from 2-month-old cultures contained 2.2-fold and 1.8-fold more Chl *b* at L0 and L12, respectively, than cells from 1-week-old cultures. Consequently, cells from cultures of all ages exhibited a similar Chl *a*/*b* ratio at both L0 and L12 (Figure 1E).

The cellular carotenoid content showed a time-dependent increase (Figure 1F). Cells from 1- and 2-month-old cultures showed 1.7-fold and 3.0-fold increases, respectively, in total carotenoid content compared to cells from 1-week-old cultures at L0. Cells from 1- and 2-month-old cultures contained 1.4-fold and 2.3-fold more total carotenoids, respectively, than cells from 1-week-old cultures at L12.

### 2.2. The Cell Division Cycle of Euglena Shows Circadian Resonance with the Light/Dark Cycle

**Figure 2:**
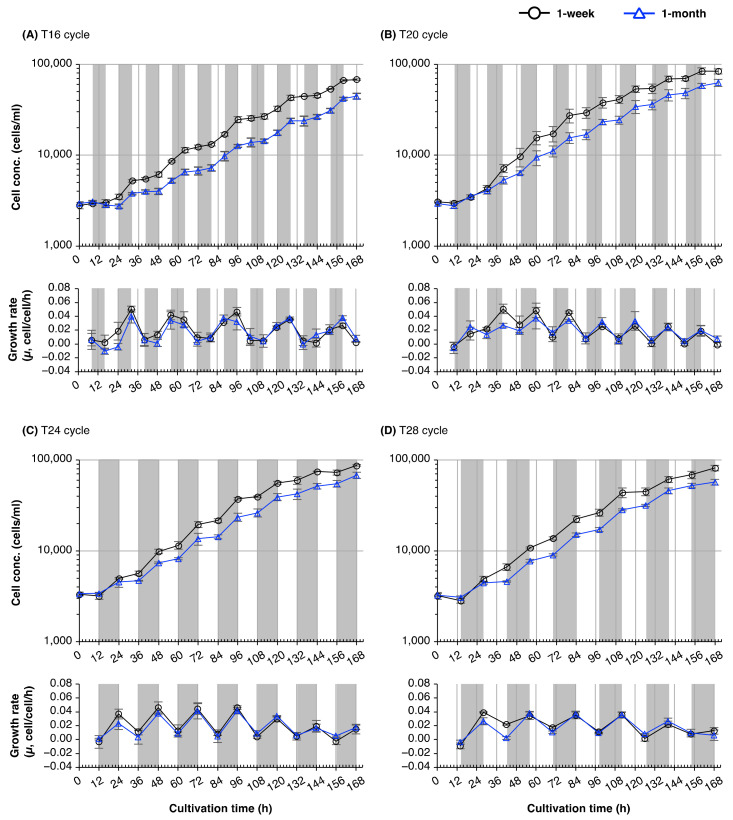
Effect of non-24-h light/dark cycles (T-cycles) on increases in cell numbers of *E. gracilis* from cultures of different ages. (**A**–**D**): Time courses of cell concentration (upper panels) and growth rate (*µ*, cell/cell/h) (lower panels) of *E. gracilis* cells from 1-week- and 1-month-old cultures under (**A**) T16; (**B**) T20; (**C**) T24; and (**D**) T28 cycles. *E. gracilis* cells were cultured under T24 cycles for 1 week or 1 month, and subsequently inoculated into fresh medium. The algal cells were then cultured under the different T-cycles for 7 days. Data are means ± SD (*n* = 3).

Synchronizing the external light/dark cycle with the internal rhythm enhances fitness in cyanobacteria and land plants [18,19]. To determine whether the circadian period of *E. gracilis* changed with culture age, we examined circadian resonance by measuring cell numbers in cultures of different ages under non-24-h light/dark cycles (T-cycles). When *E. gracilis* was grown under 8 h/8 h light/dark cycles (T16), the number of cells increased rhythmically, but the time of peak growth rate was independent of the external light/dark cycle (Figure 2A). Cultivation of cells from 1-week-old and 1-month-old cultures in T16 cycles for 7 days produced concentrations that were 17–22% and 21–29% lower, respectively, than those produced by growth for the same time in other light/dark regimes (Supplementary Figure S1). On the other hand, when cells from 1-week-old and 1-month-old cultures were grown under 10 h/10 h, 12 h/12 h, or 14 h/14 h light/dark cycles (T20, T24, and T28 cycles, respectively), the cell counts increased during the dark period, and the peak growth rate was synchronized to the external light/dark cycle (Figure 2B–D).

**Figure 3:**
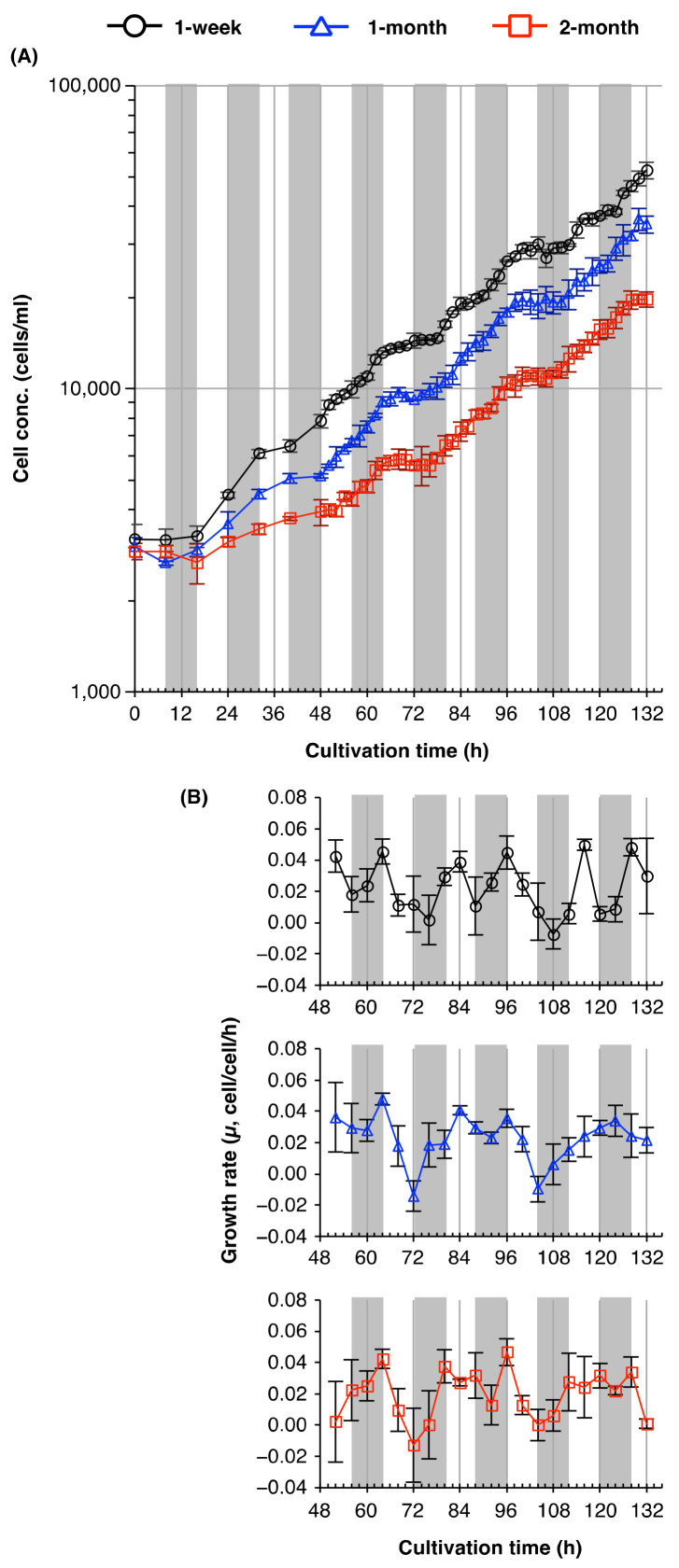
Periods of the cell division cycles of *E. gracilis* from young and old cultures under T16 light/dark cycles. Time courses of the (**A**) cell concentration and (**B**) growth rate (*µ*, cell/cell/h) of *E. gracilis* cells from 1-week-old, 1-month-old, and 2-month-old cultures under T16 cycles. *E. gracilis* cells were cultured under T24 cycles for 1 week, 1 month, or 2 months, and subsequently inoculated into fresh medium. Cells were collected every 2 h for 3.5 days from 48 h post-inoculation in order to enable measurement of cell concentration; growth rates were calculated over 4-h intervals. Data are means ± SD (*n* = 3).

To determine the period of cell division cycle under T16 cycles more accurately, we measured the time-course of changes in the cell concentrations of cultures established from 1-week-old, 1-month-old, and 2-month-old cultures. The concentration of cells from 1- or 2-month-old cultures increased rhythmically with a period of 32 h, of which 16 h (one T16 cycle) were devoted to cell division, and the remainder (another T16 cycle) to developmental growth (Figure 3A). By contrast, the concentration of cells from 1-week-old cultures increased with a different rhythm, with a period of 16–20 h—much shorter than that seen in cell populations cultured for 1 or 2 months (Figure 3A,B).

### 2.3. Cell Viability of Euglena from Young and Old Cultures

**Figure 4:**
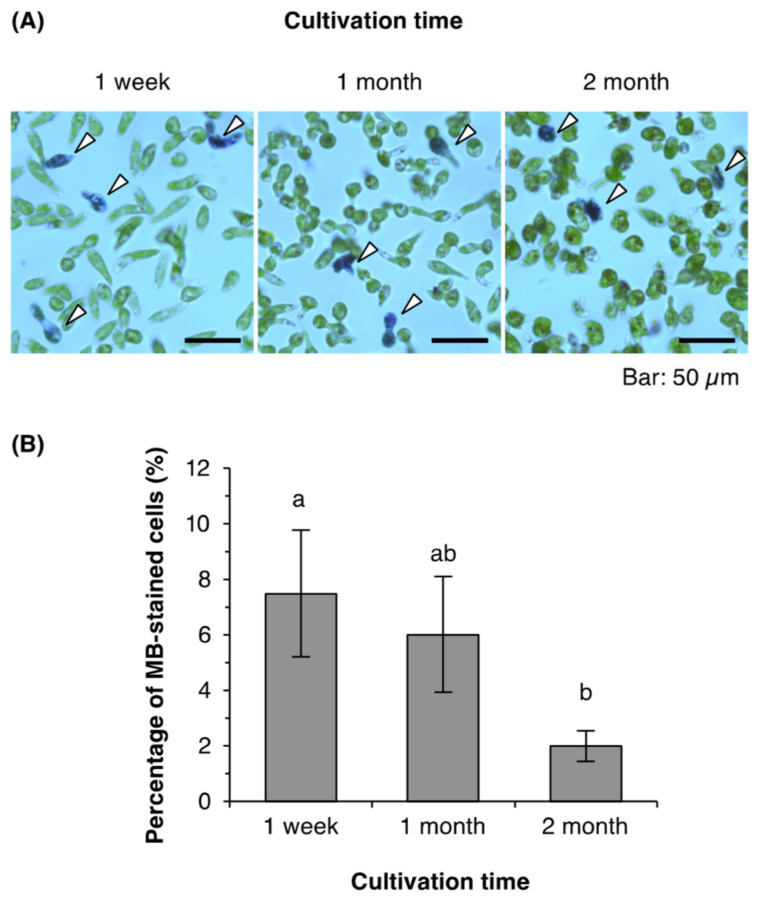
Viability of *E. gracilis* cells from young and old cultures. (**A**) Appearances of *E. gracilis* cells from 1-week-old, 1-month-old, and 2-month-old cultures stained with 0.04% methylene blue (MB). Arrowheads indicate MB-stained cells. (**B**) Percentages of MB-stained cells in the populations after washing prior to inoculation. Data are means ± SD (*n* = 3). Bars labeled with the same letter do not differ significantly from each other (Tukey’s multiple range test, *p* < 0.05).

In all T-cycle treatments, cells from 1-month-old cultures produced lower concentrations than did cells from 1-week-old cultures (Figure 2). After cultivation for 7 days, 1-month-old cultures produced cell concentrations that were 25–34% lower than those of cells from 1-week-old cultures (Figure 2; Supplementary Figure S1). We checked whether the cells from older cultures showed lower viability by performing a dye exclusion assay on the samples collected from 1-week-old, 1-month-old, and 2-month-old cultures (Figure 4). The percentage of cells stained with methylene blue (MB) did not differ significantly between 1-week-old and 1-month-old cultures (7% and 6%, respectively; Figure 4B); moreover, only 2% of cells from 2-month-old cultures were stained with MB—a lower percentage than that in cells from either younger culture.

### 2.4. Plasticity of the Circadian Rhythm of Cell Division in Young and Old Cultures of Euglena

**Figure 5:**
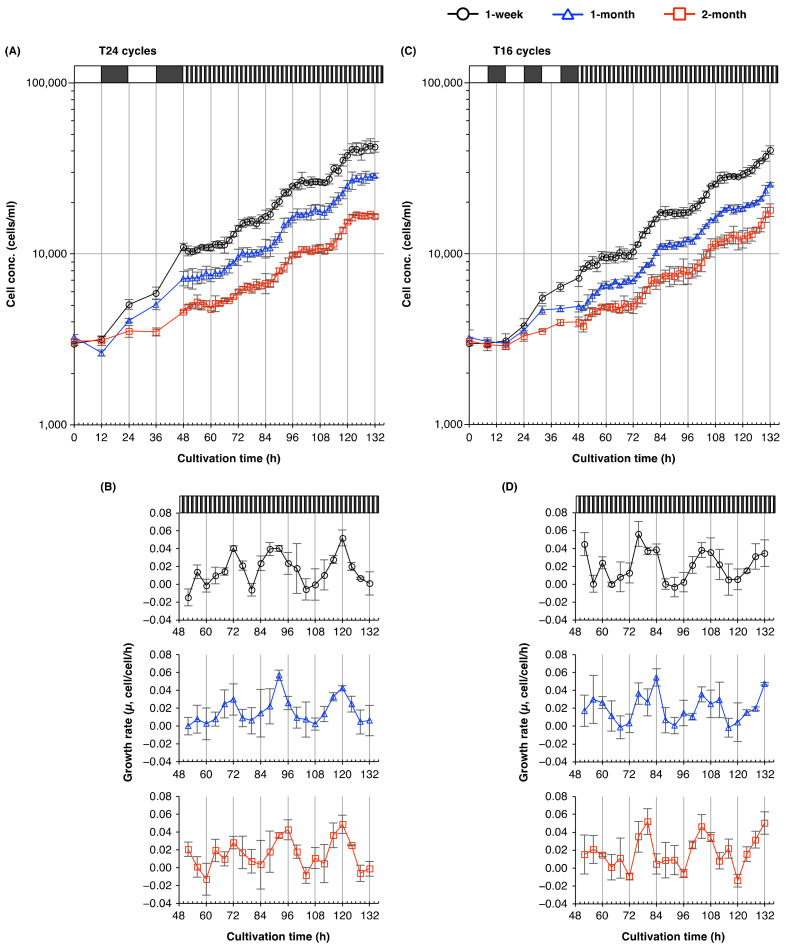
Rhythms of the cell division cycle of *E. gracilis* from young and old cultures exposed to T16 cycles. Time courses of (**A**,**C**) cell concentration and (**B**,**D**) growth rate (*µ*, cell/cell/h) of *E. gracilis* cells from 1-week-old, 1-month-old, and 2-month-old cultures under free-running conditions following entrainment to T24 and T16 cycles. *E. gracilis* cells were cultured under T24 cycles for 1 week, 1 month, and 2 months, and subsequently inoculated into fresh medium. Algal cells were cultured under T24 or T16 cycles for 48 h and then transferred to free-running conditions (1 h/1 h light/dark cycles) for 3.5 days. Data are means ± SD (*n* = 3). Growth rates under free-running conditions were calculated over 4-h intervals.

As *E. gracilis* cells from older cultures showed a longer period of cell division cycle under T16 cycles (Figure 3), we examined the period of the cell division cycle of cells from young and old cultures under free-running conditions (1 h/1 h light/dark cycles) in order to determine whether culture age affected the endogenous rhythm of this alga. The time course of the changes in cell concentration of cells from 1-week-old cultures entrained to 12 h/12 h light/dark cycles and released to free-running conditions indicated that cells divided rhythmically with a period of 24 h, of which 14 h were devoted to cell division, and the remainder to developmental growth (Figure 5A,B). Cells from 1- or 2-month-old cultures showed a similar rhythm of cell count increase.

Next, we examined whether the external light/dark regime affected the circadian rhythm of cell division cycle. Following entrainment to T16 for 2 days (3 × T16 cycles), the number of algal cells increased over the first 24 h in free-running conditions, regardless of the length of time in pre-culture (Figure 5C,D). Notably, cells entrained to T16 cycles divided during the first 12 h of the free-running treatment following the last dark period; this contrasted with the case of entrainment to T24, when cell division was delayed for 12 h until the first subjective night (Figure 5A,B).

**Figure 6:**
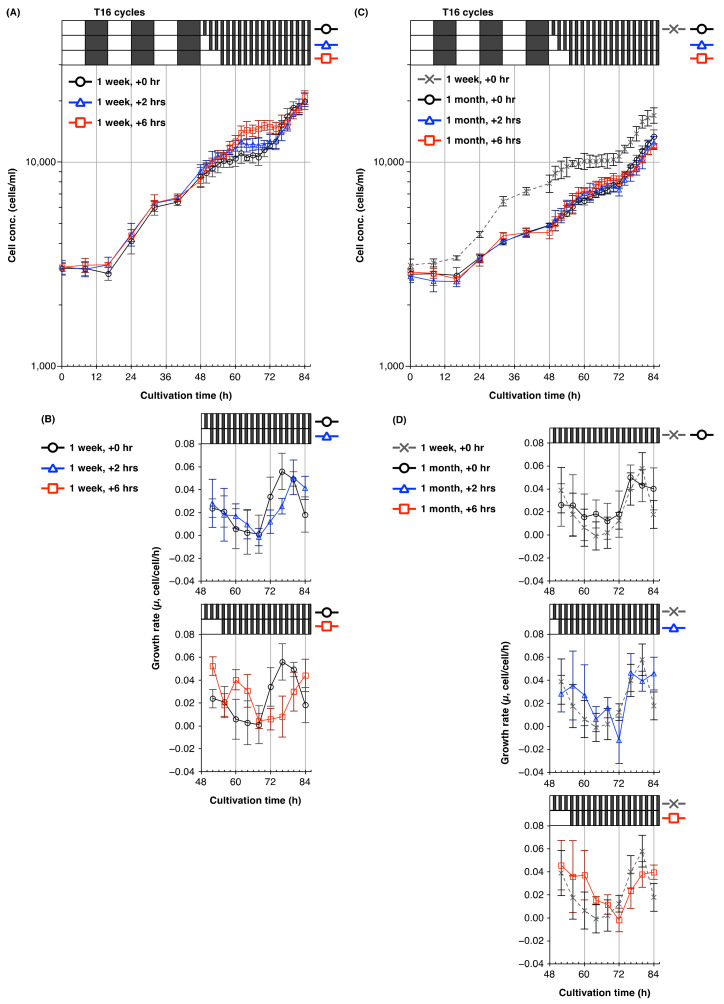
Plasticity of the period of cell division cycle of *E. gracilis* from young and old cultures. Time courses of the (**A**,**C**) cell concentration and (**B**,**D**) growth rate (*µ*, cell/cell/h) of *E. gracilis* cells from 1-week-old (**A**,**B**), and 1-month-old (**C**,**D**) cultures under free-running conditions beginning at different start times. *E. gracilis* cells were cultured under T24 cycles for 1 week and 1 month, and subsequently inoculated into fresh medium. After entrainment to 3 × T16 cycles, algal cells were irradiated for 0, 2, or 6 h, and then released to free-running conditions (1 h/1 h light/dark cycles). Data are means ± SD (*n* = 4). Growth rates under free-running conditions were calculated over 4-h intervals.

Since entrainment to T16 cycles caused a phase shift of the time of the circadian peak growth rate by 8–12 h (Figure 5A–D), we examined the effect of shifting the time of release to free-running conditions after entrainment to T16 cycles. For cells pre-cultured for 1 week prior to treatment, a delay of 6 h in the start time of the free-running conditions delayed the onset of cell division by roughly 8 h, compared with the control treatment (Figure 6A,B); a delay of 2 h in releasing cells to free-running conditions also caused a phase shift in the cell division cycle. Interestingly, the growth rate of cells pre-cultured for 1 week and given a 6 h delay showed a marked increase at the 60–64 h time period, which corresponded with the first subjective night. This increase was not observed in cells cultured for 1 month and given a 6 h delay (Figure 6C,D), although this treatment did cause a phase shift in the cell division cycle.

## 3. Discussion

### 3.1. Characteristics of E. gracilis Cells in Old Cultures

*E. gracilis* cells from 1- or 2-month-old cultures showed abnormal cell morphology, as they were gourd- or mushroom-shaped (Figure 1B). We also observed multiheaded cells in 2-month-old cultures (Supplementary Figure S2). These observations were consistent with a previous study of *E. gracilis* cell morphology in prolonged stationary phase [25]. Gomez et al. [25] observed morphological changes from elongated cells during the log phase to spherical, enlarged, or clumping cells during the stationary phase after cultivation for 360 h.

Previous studies have reported that dark orange or reddish-brown to dark brown globules are commonly observed within Euglenida cells in aged cultures [25,26]. Consistent with this, we observed several brown globules within cells from 1- or 2-month-old cultures (Figure 1B; Supplementary Figure S2). Kashiyama et al. [26] isolated the brown globules from *E. gracilis* cells, and determined that they contained chlorophyll-*a*-derived 13^2^,17^3^-cyclopheophorbides *a* enol (cPPB-*a*E) and related compounds. Since cPPB-*a*E and its derivatives gradually accumulate in *E. gracilis* cells after the stationary phase, Kashiyama et al. [26] concluded that globule formation was associated with 13^2^,17^3^-cyclopheophorbide enol (CPE)-accumulating chlorophyll catabolism (CACC) during chloroplast dismantling. Our previous studies [27,28,29] showed that although the cellular chlorophyll (*a* + *b*) content of *E. gracilis*, grown under high-intensity light or in cold and intense light conditions, decreased by up to 75% and 87%, respectively, pigmented brown globules were not observed within stressed cells. In addition, *E. gracilis* cells with abnormal morphology were not observed in cultures grown under these stressed conditions [27,28,29]. Ultrastructural analysis of *E. gracilis* in aged cultures by Kashiyama et al. [26] revealed the formation of lipid bodies in the dismantled chloroplasts, as in the senescing cells of higher plants [14]. Similarly, electron microscopy by Gomez et al. [30] showed increases in the size and numbers of osmiophilic globules in the chloroplasts of *E. gracilis* in aged cultures. The formation of brown globules within *E. gracilis* cells might therefore be age-dependent. The appearance of such globules in 1- and 2-month-old cells suggests that the dismantling of the chloroplasts or chlorophyll breakdown occurs at this stage. Accordingly, we considered 1-week-old cultures to be young cultures, and 1- or 2-month-old cultures to be aged or senescent cultures, in the following experiments.

### 3.2. Circadian Resonance of Cell Division of Euglena with Light/Dark Cycles

Aging alters the period and amplitude of circadian rhythms in a variety of organisms, including fungi, insects, mammals, and land plants [16,31,32,33,34]. Within an individual plant of *A. thaliana*, early-emerging (older) leaves have shorter circadian periods than late-emerging (younger) leaves [16]. These studies led us to speculate that the circadian rhythm in *E. gracilis* would change as culture age increased. Since several organisms exhibit a fitness advantage when the external light/dark cycle (T-cycle) matches the endogenous period (circadian resonance) [17,18,19], we examined the growth of *E. gracilis* cells from young and old cultures under different T-cycles, in order to investigate changes in the circadian rhythms of cell division under prolonged-stationary-phase conditions (Figure 2).

In T-cycle experiments, cell concentration after cultivation was significantly lower following exposure to T16 cycles for 7 days than after other treatments, for both the young and old cultures (Supplementary Figure S1). This lower growth rate is consistent with a previous report [5] that exposure to 8 h of light before transfer to continuous darkness was insufficient for cell cycle progression. *E. gracilis* cells grown in photoautotrophic conditions have three photoinduced commitment points in the cell division cycle: the G1, S, and G2 phases [35]. Hagiwara et al. [35] calculated that, in cells of *E. gracilis* incubated at 25 °C, the S phase lasted 4.8 ± 0.6 h, and the G2 + M phase lasted 3.3 ± 0.4 h. Once cells have entered the M phase, they can complete mitosis in darkness. In addition, Hagiwara et al. [5] showed that the commitment of G1, S, and G2 cells of *E. gracilis* is under circadian control; light exposure around subjective dusk was most effective at inducing commitment to cell division, whereas light around subjective dawn was not effective. In the current study, *E. gracilis* from old cultures appeared unable to achieve the photoinduced commitment to undergo mitosis within the light period of each T16 cycle, although cells from 1-week-old cultures could do so. Our T-cycle experiments suggest either that *E. gracilis* cells from 1-week-old cultures reach developmental maturity sooner than cells from 1- or 2-month-old cultures, or that the period of the cell division cycle is more plastic in response to the external light/dark cycle in young cells of this alga.

T-cycle experiments (Figure 2) suggested that T16 cycles were considerably dissonant with the endogenous circadian periods of *E. gracilis*. To reveal the plasticity of the period of the cell division cycle, and responsiveness to external light/dark regimes, we examined the rhythmic period of the cell division cycles of cultures established from 1-week-old, 1-month-old, and 2-month-old cultures under T16 conditions in detail. Under T16 cycles, the growth curve of cells from 1- and 2-month-old cultures showed a stepwise increase every 32 h (Figure 3). This periodicity of the cell division cycle was consistent with a previous study [23] that reported the *E. gracilis* cells divided with a period of ≈33 h under T16 conditions. Edmunds and Funch [23] suggested that this might result from either a free-running circadian rhythm or entrainment to a 32-h cycle by frequency demultiplication (skipping of a cycle). Cells from 1-week-old cultures clearly showed more peaks or troughs in their growth rates compared with cells from older cultures under T16 cycles. This result suggests that the cell populations established from 1-week-old cultures had a shorter circadian period of their cell division cycles under T16 cycles. Since the old medium was washed off before inoculation, we could rule out the possibility that differences in the rhythmic periods of the cell division cycles and growth rates between cells cultured for different lengths of time resulted from exogenous factors, such as the accumulation of waste metabolites, or nutrient depletion in the medium.

### 3.3. Viability of Euglena Cells from Young and Old Cultures

*E. gracilis* cells from older cultures produced lower cell concentrations under T-cycles and free-running conditions when inoculated into fresh medium (Figure 2, Figure 3, and Figure 5). Dye exclusion assays excluded the possibility that this decrease resulted from higher concentrations of dead cells in the populations of older cells used for the inoculation (Figure 4), as the percentage of MB-stained cells in 2-month-old cultures was in fact rather lower than those in 1-week-old or 1-month-old cultures. This suggests that *E. gracilis* cells of different culture ages differ in their capacity for cell division.

### 3.4. Plasticity of Circadian Rhythms of Cell Division in Euglena from Young and Old Cultures

Aging shortens the free-running circadian period in mammals and land plants [16,36]. By contrast, under free-running conditions (1 h/1 h light/dark cycles), *E. gracilis* cells divided with a period close to 24 h, regardless of culture age (Figure 5). This suggests that *E. gracilis* has a robust endogenous circadian rhythm, whose free-running period is resistant to aging. This result, together with our T-cycle experiments, suggests that *E. gracilis* cells from 1-week-old cultures—which showed a period of their cell division cycle partly matched to T16 cycles (Figure 3)—have a higher circadian plasticity or responsiveness to external light/dark regimes than cells from older cultures.

Mathematical and experimental studies by Dodd et al. [37] demonstrated temporal plasticity in the circadian period of *A. thaliana* when the free-running period and T-cycle were dissonant. Plasticity of circadian rhythms of locomotor activity has also been reported in insects and mammals [38,39,40]. Aton et al. [38] showed that the behavioral period of young mice was significantly shorter than that of older mice (>3 months) after entrainment to short T-cycles (T20), and suggested that aging diminished the ability to entrain to photic stimuli. In line with such studies, the peak growth rate of *E. gracilis* cells from 1-week-old cultures exposed to T16 cycles and transferred to free-running conditions after 6 h of light irradiation occurred at a time corresponding to the first subjective night (Figure 6A,B), which may result from circadian plasticity. This suggests that young cells of *E. gracilis* can “remember” non-24-h light/dark cycles and anticipate the time of dusk so as to regulate nocturnal events such as cell division, whereas older cells of this alga have a shorter “memory” of the external light regime, and easily and immediately revert their cell division period to its steady-state free-running period close to 24 h. The differences in circadian plasticity or responsiveness to external light/dark regimes may not be attributed solely to chronological aging of the algal cells; in the prolonged-stationary culture, *E. gracilis* cells would be exposed to multiple stressors, such as self-shading of light, metabolite accumulation, drop in pH, and nutrient depletion. These stressors may have induced acclimation responses such as metabolic reprogramming and cell cycle arrest, which ultimately increase the chances of population survival under unfavorable environmental conditions in the prolonged-stationary algal cells.

When *E. gracilis* cells were transferred to free-running conditions immediately after the last dark period of a T16 cycle, we did not observe obvious alterations to the period of the cell division cycle in any of the culture ages examined (Figure 5C,D). In *A. thaliana* plants entrained to dissonant T-cycles, the oscillator period returns to the steady-state period within 40–55 h, whereas, in animals, alterations to the circadian period persist for at least several months [37,39,40]. Our results suggest that any alteration of the rhythmic period of the cell division cycle of this alga caused by entrainment to the environmental period reverts easily and immediately to its steady-state free-running period close to 24 h. For further understanding of the plasticity and robustness of the circadian rhythm of *E. gracilis*, it would be necessary to monitor cell count increase over an interval of at least 2–4 h during entrainment to T-cycles, as well as under free-running conditions, for several days. Combinational T-cycle treatments, such as shifting from T20 cycles to T28 cycles, would also provide insight into the circadian plasticity of this alga.

## 4. Materials and Methods

### 4.1. Biological Materials

*E. gracilis* Klebs (NIES-48) was obtained from the Microbial Culture Collection of the National Institute for Environmental Studies (NIES) (Tsukuba, Japan). *E. gracilis* cells were grown in an incubator (Bio 2000-L; Vision Scientific, Daejeon, Korea) and illuminated at 50 µmol photons m^–2^ s^–1^ using fluorescent lamps (FL20SD/18; City Lighting, Incheon, Korea).

Algal cells were cultured in 100 mL of Cramer–Myers medium [41] in a 300-mL conical flask on a rotary shaker (SHO-2D; Daihan Scientific, Wonju, Korea) at 90 rpm under 12 h/12 h light (50 µmol photons m^–2^ s^–1^)/dark cycles. Algal cultures were maintained by inoculating cells from a 1-week-old culture into fresh medium, at an initial cell concentration of 3 × 10^3^ cells mL^–1^ every week. To prepare aging cultures, algal cells were cultured for 1 or 2 months without any replacement or supplementation of the medium.

### 4.2. Time-Course Analysis of Euglena Cell Division in Young and Old Cultures

To analyze the time course of cell growth, cells were collected by centrifugation (1000× *g*, 2 min) after 1 week, 1 month, or 2 months in culture, and washed twice in a few milliliters of fresh medium. Cells were then inoculated into fresh medium at 3 × 10^3^ cells mL^–1^. For T-cycle experiments, the pre-cultured cells were transferred to 8 h/8 h, 10 h/10 h, 12 h/12 h, and 14 h/14 h light/dark cycles (T16, T20, T24, and T28 cycles, respectively) for 7 days.

For analyses of the internal rhythm of the cell division cycle, inoculated cells were entrained to T16 or T24 cycles for 2 days (i.e., 3 × T16 cycles or 2 × T24 cycles) and then cultured under free-running conditions (1 h/1 h light/dark cycles) for 3.5 days. For analyses of the plasticity of the internal rhythm of the cell division cycle, inoculated cells entrained to T16 or T24 cycles for 2 days were released to free-running conditions after irradiation for 0, 2, or 6 h following the last dark period. Cell concentration was measured using a plankton counter slide (MPC-200; Matsunami Glass, Osaka, Japan) under a bright-field microscope. The cell growth rate was calculated using the following equation:

Growth rate (µ, cell/cell/h) = ln(N_2_-N_1_)/(t_2_-t_1_), (1)

where N_2_ and N_1_ are the cell concentration at times t_2_ and t_1_, respectively.

In this study, cell division refers to increase in cell numbers as evaluated by the cell concentration in the culture medium. The period of cell division refers to the length of time for which the cell counts increase in a single cycle of cell division. The rhythmic period of cell division cycle refers to the interval between peaks or troughs of the growth rate curve.

### 4.3. Determination of Photosynthetic Pigment Content

To determine the cellular chlorophylls (Chls) and total carotenoid content, approximately 1 × 10^7^ cells were harvested by centrifugation (3000× *g*, 5 min) after 1 week, 1 month, or 2 months in culture. Cell samples were weighed to calculate cell fresh weight, frozen rapidly in liquid nitrogen, and stored at −80 °C until analysis. Total pigments were extracted from approximately 1 × 10^7^ cells using 4 × 1 mL buffered aqueous 80% acetone. Chl *a* and *b* concentrations in the pooled extracts were determined using the extinction coefficients, as described by Porra et al. [42]. The total carotenoid content (µg mL^–1^) of the extract was determined using the following coefficient:

Carotenoid (µg mL^−1^) = *A_480_* + 0.114*A_663_* − 0.638*A_645_* (2)

### 4.4. Dye Exclusion Assay

For the dye exclusion assay, cells cultured for 1 week, 1 month, or 2 months were washed twice with fresh medium, as described above. Approximately 2 × 10^6^ cells were suspended in 1 mL of fresh medium and stained with 0.04% MB at room temperature for 30 min. The concentration of MB-stained cells (A) was measured using a plankton counter under a microscope, before the cells were fixed in 0.05% glutaraldehyde, and the total cell concentration in the sample (B) was measured to enable calculation of the percentage of MB-stained cells (A/B × 100%).

### 4.5. Statistical Analysis

Data are expressed as mean ± standard deviation (SD). Student’s *t*-test and Tukey’s multiple range test were performed to identify statistical differences (*p* < 0.05) between and among treatments, respectively.

## 5. Conclusions

Our observations suggest that the period of circadian rhythms and responsiveness of younger cells of *E. gracilis* are more plastic and better able to match their endogenous period to external light regimes. Cells of this alga established from aged cultures had a shorter memory of external light/dark regimes. These differences in the responsiveness of the cell division period to external light conditions might result from resource partitioning, or enable survival under fluctuating light environments by preventing simultaneous cell division across a population of *E. gracilis*. Further studies are needed in order to elucidate the aging of *E. gracilis* and the ecological meaning underlying the plasticity of the circadian rhythm of cell division of this alga—especially at a single-cell level, in the absence of external stressors.

## Data Availability

The data that support the findings of this study are included in this published article and its supporting information files.

## Supplementary Materials

The following are available online at https://www.mdpi.com/article/10.3390/plants10071475/s1, Figure S1: Final cell concentrations of *E. gracilis* from 1-week-old and 1-month-old cultures following cultivation under various T-cycle treatments for 7 days. Data are means ± SD (*n* = 3). Bars labeled with the same letter do not differ significantly from one another (Tukey’s multiple range test, *p* < 0.05); Figure S2: *E. gracilis* cells with abnormal appearances. Multiheaded cells (arrowheads) were observed in 2-month-old cultures. L0 and L12 indicate the beginning and end of the light period, respectively.
