# Supplementary figures and images for: The Cell Division Cycle of Euglena gracilis Indicates That the Level of Circadian Plasticity to the External Light Regime Changes in Prolonged-Stationary Cultures
Source: Plants (Basel). 2021 Jul 19;10(7):1475. doi: 10.3390/plants10071475 (PMC8309271; doi:10.3390/plants10071475)

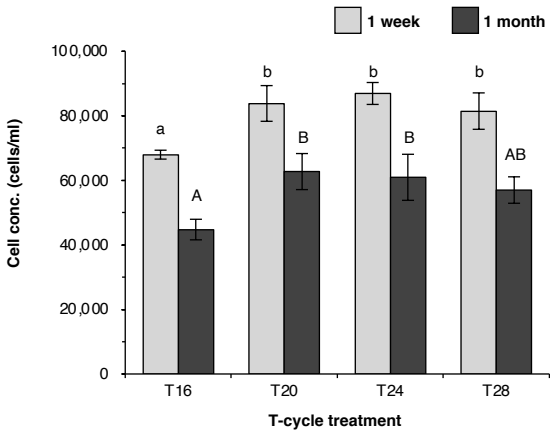

Supplement: Supplementary file 1 [file plants-10-01475-s001.zip › supp/FigS1.pdf]

L0

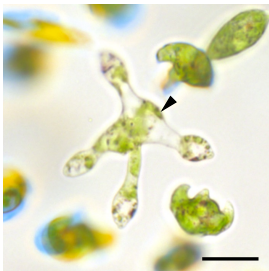

L12

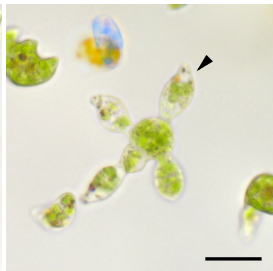

Bar: 20  $\mu\text{m}$

Supplement: Supplementary file 1 [file plants-10-01475-s001.zip › supp/FigS2.pdf]
